# The Relationship between Language Control, Semantic Control and Nonverbal Control

**DOI:** 10.3390/bs10110169

**Published:** 2020-11-06

**Authors:** Teresa Gray

**Affiliations:** Department of Speech, Language and Hearing Sciences, Graduate College of Education, San Francisco State University, San Francisco, CA 94132, USA; teresag@sfsu.edu

**Keywords:** bilingual aphasia, inhibitory control, cognitive control, resistance to distractor interference, bilingual language control

## Abstract

The purpose of this study was to examine the relationship between language control, semantic control, and nonverbal control in bilingual aphasia. Twelve bilingual adults with aphasia (BPWA) and 20 age-matched bilingual adults (AMBA) completed a language control task, semantic control task, and nonverbal control task, each designed to examine resistance to distractor interference. AMBA and BPWA exhibited significant effects of control on all tasks. To examine efficiency of control, conflict magnitudes for each task and group were analyzed. Findings revealed that AMBA exhibited larger conflict magnitudes on the semantic control task and nonverbal control task compared to the language control task, whereas BPWA exhibited no difference in conflict magnitudes between the language control task and semantic control task. Further analysis revealed that BPWA semantic control conflict magnitude was smaller than AMBA semantic control conflict magnitude. Taken together, these findings suggest that BPWA present with diminished effects of semantic control. In the final analysis, conflict magnitudes across tasks were correlated. For AMBA, semantic control and nonverbal control conflict magnitudes were significantly correlated, suggesting that these two types of control are related. For BPWA, language control and nonverbal control conflict magnitudes were significantly correlated; however, this finding may capture effects of domain general cognitive control as a function of increased cognitive load, rather than domain general cognitive control as a function of language control.

## 1. Introduction

As the population ages, the prevalence of stroke and, consequently, aphasia increases. A key feature of aphasia is impaired lexical access, indicating that activation of the target word or inhibition of the lexical competitor is disrupted. In bilingual aphasia, there is an additional factor of language control (i.e., activating the target language and inhibiting the non-target language). Currently, the relationship between lexical access and language control in bilingual persons with aphasia (BPWA) is unclear. To best help BPWA improve their ability to successfully access target words during everyday communication, we need to better understand how they manage language control.

An exploration of language control in bilingual aphasia would be incomplete without taking into account semantic control and nonverbal control. Semantic control is the ability to select one linguistic item, e.g., apple, while suppressing its semantic competitors, e.g., orange, cherry, and strawberry. Nonverbal control, sometimes referred to as cognitive control and executive function, is the ability to manage nonverbal information and is associated with a variety of functions, e.g., attention, memory, and mental flexibility. In the present study, the relationships between these three constructs in BPWA and age-matched bilingual adults (AMBA) is examined in efforts to better understand the relationship between these three processes and how they present in bilingual aphasia.

### 1.1. Language Control

In the bilingual brain, two languages are active simultaneously. This has been demonstrated in bilinguals when speaking [1,2,3,4] and reading [5,6,7]. In a seminal study, Herman and colleagues [3] employed a bilingual picture-word interference task to investigate whether the non-target language is activated during lexical access of the target language. Participants were asked to name pictures in English and ignore distractor words that were presented in English or Dutch. The pictures were divided into four categories: phonologically related to the target, semantically related to the target, unrelated to the target, or phonologically related to the Dutch name of the target. Findings revealed that lexical items from the non-target language (Dutch), were activated during naming in English, offering support for simultaneous language activation in bilinguals.

In the monolingual brain, one language is represented, meaning that lexical nodes and phonemes from one lexicon are active. However, in the bilingual brain, since two languages are simultaneously active, lexical nodes and phonemes from two (or more) languages are activated in parallel. Thus, for the target word to be selected, the system must manage activated nodes from different languages in order to resolve the conflict and select the target. One of the foremost models of bilingual language control is the Inhibitory Control (IC) model [8]. The IC model posits that to resolve the competition between language task schemas, the non-target language is inhibited while lexical items from the target language are activated and then selected. The IC model accounts for various bilingual language processing situations, such as codeswitching and translation, which are two distinct processes [8].

Codeswitching requires that an individual switch from one task schema to another [9,10,11]. Meuter and Allport [10] examined codeswitching in 16 bilingual, healthy adults. Participants were asked to name numerals in L1 (the dominant language) and L2 (the non-dominant language) in switch and non-switch conditions. Based on previous studies that examined switching between dominant and weaker tasks, the authors predicted an asymmetrical switch cost: switching into L1 would incur longer response times than vice versa (switching into L2). This is because naming in the non-dominant L2 requires active inhibition of the dominant L1, which is more effortful than inhibiting the non-dominant L2 to name items in L1. This increased effort results in difficulty releasing the active inhibition in order to switch into L1. Thus, inhibition of L1 continues into the initial stages of the next trial, creating negative priming of the L1 lexicon. Meuter and Allport’s [10] results highlighted the expected asymmetrical switch cost and offer support for the notion that the IC model accounts for how two languages are activated and inhibited in codeswitching.

Language control is also critical for translation. The Revised Hierarchical Model (RHM) [12] and mixed model [13] have been used to explain patterns of bilingual lexical access and translation processes. In the RHM, the semantic system has bidirectional links to the first language (L1) and second language (L2), and the weight of each link is a function of proficiency. As proficiency strengthens, so does the link from the word form to the semantic system. There are also bidirectional connections between L1 and L2. If L2 is the non-dominant language, the L2-L1 link is stronger than the L1-L2 link. When translating a word from L1 to L2, the target is named in L2 and the L1 word is successfully inhibited. During this process, the L1 lemmas related to the L1 target word are also activated and compete for selection. According to the IC model, the lemmas with an L1 tag are suppressed so that only lemmas with an L2 tag compete for output. Thus, the IC model accounts for patterns observed in translation performance, which requires some degree of language control. Clearly, bilinguals are constantly managing two languages, whether translating or switching between two languages or speaking in the target language and inhibiting the non-target language.

### 1.2. Semantic Control

Another type of control required for lexical access, regardless of whether one is monolingual or bilingual, is semantic control. During word retrieval, the target concept is activated, along with competitors with similar semantic features [14]. The interactive model [15] posits that semantic, lexical, and phonological representations compete for selection through a combination of spreading activation and feedforward and feedback connections that prime activation throughout the lexical network. Thus, lexical selection involves competition at semantic and phonological levels [16,17,18].

Numerous studies have examined semantic interference in picture word interference paradigms [16,19,20,21,22,23,24,25], word translation [26,27], and blocked cyclic naming [17,28,29], finding that adults with aphasia and age-matched controls are slower to name stimuli flanked by semantically related items compared to unrelated items. Schnur and colleagues [17] examined the semantic blocking effect in monolingual adults with aphasia and age-matched controls. Participants were asked to name pictures presented in four blocked-cyclic homogenous (semantically related) and mixed (semantically unrelated) sets. In each cycle, the set items remained constant but the order was presented differently, inducing activation and competition among word items. Related distractors are primed and activated by the targets, but the targets are selected because they receive more activation. Results revealed that both groups exhibited semantic interference in homogenous sets: naming items in the homogenous sets took longer than naming items in the mixed sets. The authors also performed an error analysis to determine if outcomes were caused by “too much excitation” or “too much inhibition.” Because semantic substitutions were observed during homogenous sets, authors concluded that semantic interference was caused by over-excitation of lexical-level competitors. More recently, Calabria et al. [29] examined the performance of highly proficient Catalan-Spanish bilingual adults with aphasia and age-matched controls on a blocked cyclic naming task. Participants were asked to name line drawings presented in homogenous and mixed sets across four cycles. The word sets were within-language and presented in Catalan and Spanish. Results revealed that both groups exhibited semantic interference on the homogenous sets and that the adults with aphasia exhibited larger interference effects compared to the control group. Interestingly, the larger semantic interference effect observed in patients was exaggerated in their non-dominant language on the homogenous set, indicating that two languages may differ in their relationship to semantic control. It is evident that persons with aphasia are still susceptible to semantic interference and the effect is more extreme compared to age-matched counterparts.

### 1.3. Nonverbal Control

Cognitive control is a collection of processes that are used to regulate a variety of functions, e.g., reasoning, planning, and goal directed behavior [30,31,32]. It consists of proactive control, which anticipates change, and reactive control, which responds to change [33,34]. It has been argued that conflict monitoring is an integral component of cognitive control because it aids in the monitoring of interference that may arise from task demands [35,36]. In addition, inhibition-related functions (i.e., inhibition and interference control) are critical variables to consider when investigating cognitive control because they involve the ability to resist or resolve interference. Over the years, a thorough interest in these functions has emerged. Various taxonomies have been proposed [37,38,39] and there is a large body of work that uses various experimental paradigms to examine inhibition [40,41,42,43]. Critical to the current discussion are the inhibition-related functions established by Friedman and Miyake [44] because they distinguish between three inhibition-related functions: (1) *prepotent response inhibition*, which is the suppression of an automatic response (e.g., the Stroop task [45]); (2) *resistance to distractor interference*, which is the suppression of stimuli that are simultaneously competing for selection (e.g., the Flanker task [46]); and (3) *resistance to proactive interference*, which is suppression of the stimulus that was previously the target (e.g., Cued recall [47]). Friedman and Miyake’s [44] three constructs are valuable because they offer a systematic structure for the examination of verbal control and nonverbal control, resulting in meaningful comparisons across domains.

The Flanker task [46] is an example of Friedman and Miyake’s [44] resistance to distractor interference. During the Flanker task, a row of arrows is presented. The target arrow is red and non-target, flanking arrows are black. The participant is instructed to pay attention to the red arrow and must inhibit the distracting, flanking arrows that compete for selection. If all arrows are facing the same direction, this is considered a congruent trial and has been shown to evoke higher accuracies and faster response times compared to the incongruent condition, when the target arrow is facing the opposite direction than the non-target arrows. This difference between congruent and incongruent trials is called the “congruency effect.” Resistance to distractor interference is most similar to language control: both contexts require the suppression of non-target stimuli that are competing with the target for selection.

### 1.4. Relationships between Language Control, Semantic Control, and Nonverbal Control

A large body of work supports an overlap between language control and nonverbal control processes [48,49,50,51]. Costa, Hernandez, and Sebastian-Galles [51] compared the performance of 100 monolingual adults and 100 bilingual adults on the attention network task (ANT). The ANT employs a Flanker task that includes alerting and orienting cues. Response times varied based on cue types. Bilingual response times were faster than monolingual response times. Bilinguals were also more efficient when responding to the alerting cue condition, resolving conflict, and switching between trial types. These findings suggest that bilingual adults are more adept at managing nonverbal control compared to their monolingual counterparts, suggestive of some cross-talk between verbal and nonverbal control mechanisms. However, notably, another body of work has found no evidence of healthy bilingual adults outperforming healthy monolingual adults on nonverbal cognitive control tasks [52,53,54,55], suggesting no overlap between language control and nonverbal control.

Recent work in bilingual aphasia has examined the relationship between language control and nonverbal control [29,56,57,58,59,60,61]. These studies have used a variety of tasks (e.g., the Flanker task, the Stroop task, lexical decision, fluency) that examine different types of inhibition related functions. Some studies reveal an overlap between language control and nonverbal control mechanisms, whereas others have not. For instance, Green et al. [60] employed conflict ratios to examine control patterns in two BPWA and AMBA. Conflict ratios identify proportional changes as a function of conflict, enabling researchers to examine how much effort it takes to resolve conflict on a given task. They are found by taking the difference between the performance on a conflict trial and non-conflict trial and dividing that by the performance on the non-conflict trial. Participants completed two verbal control tasks (the Stroop task and lexical decision) and one nonverbal task (the Flanker task). Results revealed that compared to 12 AMBA, patient 1 (with subcortical damage) was less efficient at resolving conflict on the verbal tasks, as demonstrated by large conflict ratios. However, this patient exhibited no evidence of conflict on the Flanker task, resulting in a small conflict ratio. Patient 2 (with parietal brain damage) was less efficient at resolving conflict on the Flanker and lexical decision task, as demonstrated by large conflict ratios. Performance on the Stroop task was within normal limits for L1 (accuracy and response times) and L2 (reaction times) but L2 accuracy was marked by a large conflict ratio. These results underscore the importance of patient variability regarding verbal control and nonverbal control abilities. More recently, Gray and Kiran [58] examined verbal and nonverbal resistance to distractor interference in 13 Spanish-English BPWA and 20 AMBA. AMBA exhibited congruency effects on both a verbal and nonverbal triad task, whereas BPWA exhibited the congruency effect only on the nonverbal triad task. Conflict ratios were also examined. AMBA exhibited verbal and nonverbal conflict ratios that were both significantly above zero, whereas for BPWA, only nonverbal conflict ratios were significantly above zero. According to the authors, it could be that a small conflict ratio may be reflective of overall slowed lexical processing: BPWA are slow on the congruent condition, which contributes to a smaller conflict ratio. Secondary analyses revealed that for only AMBA, verbal and nonverbal conflict ratios were significantly correlated, suggesting an overlap in language control and nonverbal cognitive control mechanisms, whereas BPWA results were not significant, suggesting no overlap in these control mechanisms. These findings highlight a dissociation between how AMBA and BPWA manage control across language and nonverbal domains. Also in 2016, Faroqi-Shah [57] evaluated how monolingual adults without aphasia, bilingual adults without aphasia, monolingual persons with aphasia, and BPWA performed on a Stroop task and Flanker task. Most pertinent to the current discussion is that BPWA exhibited the congruency effect on the Stroop task and Flanker, suggesting that patients are able to resolve conflict in tasks requiring language control and nonverbal control. Notably, BPWA exhibited larger conflict magnitudes (i.e., the difference between congruent and incongruent conditions) compared to their healthy counterparts, suggesting that BPWA are less efficient at resolving conflict. To summarize, while there is some evidence that BPWA (1) exhibit effects of conflict on both verbal and nonverbal tasks and (2) do not exhibit an overlap between language control and nonverbal control mechanisms, we still do not fully understand the complex relationship between these mechanisms. While most work is focused on language control and nonverbal control, there is a third variable, semantic control, that receives little attention.

Few studies have examined semantic control in relation to language control and nonverbal control in bilingual populations. For a bilingual individual to retrieve a word, the non-target language must be inhibited, as well as semantic competitors. Thus, it is logical to account for semantic control when investigating verbal and nonverbal control mechanisms. In the Calabria et al. [29] study discussed above, the authors examined nonverbal control in addition to semantic control in 11 Catalan-Spanish BPWA and 13 Catalan-Spanish AMBA. Recall that participants completed a semantic blocked cyclic naming task with BPWA exhibiting larger effect of semantic interference in their non-dominant language compared to the dominant language, suggesting that the two languages are differentially affected by semantic competitors. Participants also completed a nonverbal Flanker task. The authors identified a positive correlation between the conflict cost (i.e., congruency effect) on the effect of semantic interference and the conflict cost on the Flanker task for the non-dominant language only. This finding suggests a partial overlap between semantic control and nonverbal control, offering compelling evidence for further studies to examine this unique relationship.

In the present study, the relationship between language control, semantic control, and nonverbal control was investigated. To do this, the performance of AMBA and BPWA was examined on three triad tasks designed to examine resistance to distractor interference, based on Friedman and Miyake’s [44] model of inhibition-related functions. The experimental paradigms are designed so that one measures language control, one measures semantic control, and one measures nonverbal control. Like the well-known Flanker task, the language and nonverbal control tasks include congruent and incongruent conditions, require the active suppression of non-target stimuli, and are designed to capture the congruency effect. The semantic control task includes unrelated and related conditions that correspond to congruent and incongruent conditions. The related condition is more difficult and requires more effort to resolve conflict compared to the unrelated condition, resulting in the “relatedness effect,” captured by longer and/or less accurate response times. To better understand the relationships among these three types of control, the following questions were asked.

Do AMBA and BPWA exhibit the congruency effect on language control and nonverbal control tasks and the relatedness effect on the semantic control task?

Based on previous studies that examined the performance of bilingual persons without aphasia on nonverbal control and semantic control tasks [17,48,49], AMBA are expected to exhibit both the congruency effect and relatedness effect. Based on previous studies that examined BPWA performance on nonverbal control and semantic control tasks [29,58,59,60,61], BPWA are expected to exhibit the congruency effect on the nonverbal control task and relatedness effect for the semantic control task. Similar to AMBA, BPWA exert more effort to suppress distracting information on an incongruent condition compared to a congruent condition on a nonverbal task, and the semantic network in BPWA is susceptible to semantic interference that would evoke slower and/or less accurate responses on the related condition that includes semantically related distractors. Predicted outcomes for BPWA on the language control task are unclear. According to Gray and Kiran [58] and Green et al. [60], BPWA should not exhibit the congruency effect; however, based on Faroqi-Shah et al. [57], BPWA should exhibit the congruency effect on the language control task.

Is the efficiency of inhibition on the language control, semantic control, and nonverbal control tasks different between groups?

Efficiency of inhibition was investigated by measuring conflict magnitude for each task for each group. Instead of using the conflict ratio that takes a proportion of the conflict cost, the conflict magnitude (i.e., the difference between conflict and non-conflict trials) was employed because it captures the amount of effort required to inhibit distracting non-target stimuli and resolve conflict. Based on Faroqi-Shah et al. [57], it isexpected that BPWA will be less efficient at inhibiting the non-target language on the language control task compared to AMBA, demonstrated by a large conflict magnitude. If BPWA do not show the congruency effect (i.e., no evidence of interference) on the language task, then it is expected that BPWA will present with conflict magnitudes that are smaller than AMBA conflict magnitudes. Based on previous studies that have examined semantic control in patient populations [17,29,59], it is expected that BPWA will be less efficient at inhibiting semantic distractors compared to AMBA, as demonstrated by a larger conflict magnitude. Based on Green et al. [60] and Dekhtyar et al. [62], it is expected that BPWA will be less efficient at resolving conflict on the nonverbal control ask compared to AMBA, as demonstrated by larger conflict magnitudes.

## 2. Materials and Methods

### 2.1. Participants

Twelve Spanish-English BPWA (mean age = 51; *SD* = 11) and 20 Spanish-English AMBA (mean age = 53; *SD* = 11) who were matched on age (*t*(30) = 0.63, *p* = 0.52) and education, (*t*(30) = −1.18, *p* = 0.24) were recruited from the San Francisco Bay Area to participate in this study. All BPWA were at least 12 months post-injury: 10 experienced a stroke and two experienced a traumatic brain injury. AMBA reported no history of neurological, cognitive, or psychological impairment. Participants were right-handed and gave informed consent according to the San Francisco State University Human Subjects Protocol.

All participants completed the Language Use Questionnaire (LUQ) [63] that accounts for language use and exposure across the lifetime. The Language Use Questionnaire specifically records the following aspects of language: *age of acquisition* for first language (L1) and second language (L2); *lifetime exposure* for hearing, speaking, and reading L1 and L2; *current exposure* for receptive (hearing) and expressive (speaking) in L1 and L2 during weekday and weekend activities (BPWA completed separate current exposure ratings for pre-injury and post-injury time points); *language proficiency* of nuclear family members; *education history* regarding language of instruction and language preference during school activities, accounting for elementary school, high school, and university; and *language ability rating* (LAR), which is an average percentage value for L1 and L2, identifying each participant’s self-rating using a 5 point scale (1 represents non-fluent skills and 5 represents native or near native skills) for casual and formal conversation contexts, in addition to reading and writing. For BPWA, the LUQ included questions that accounted for pre- and post-injury LAR. LAR has been shown to be a reliable index of language proficiency [59,64,65].

Participants were considered bilingual if they spoke two languages on a daily basis [66]. All AMBA were sequential bilingual language learners. Spanish was the first language for 18 AMBA and the average English age of acquisition (AoA) was in the teenage years. At the time of testing, four AMBA were English dominant, seven AMBA were Spanish dominant, and nine AMBA were balanced bilinguals. Eleven BPWA were sequential bilingual language learners and one BPWA was a simultaneous bilingual language learner. Spanish was the first language for nine BPWA and the average AoA for English was in childhood or the teenage years, but two BPWA had an AoA for English that was 25 and 50 years of age, respectively. Before the brain injury, five BPWA were English dominant, one BPWA was Spanish dominant, and six BPWA were balanced bilinguals. At the time of testing, six BPWA were English dominant, three BPWA were Spanish dominant, and three BPWA were balanced bilinguals. Seven BPWA experienced parallel language loss and five BPWA experienced differential language loss, losing more language in Spanish or English. (See Table 1 and Table 2.)

### 2.2. Diagnostic Testing

All BPWA completed a battery of language and cognitive diagnostic testing. The Pyramids and Palm Trees Test Picture Version (PPT) [67] determined the integrity of the semantic system; the Boston Naming Test (BNT) [68] given in both languages identified confrontation naming ability at the word level in Spanish and English; the Bilingual Aphasia Test (BAT; https://www.mcgill.ca/linguistics/research/bat) and the BAT Part C characterized receptive, expressive, and translation deficits in Spanish and English; the Cognitive Linguistic Quick Test (CLQT) [69] was administered in each patient’s dominant language and used to identify general cognitive processing, language skill, and problem solving.

As demonstrated by the PPT, BPWA demonstrated intact semantic systems. Based on the CLQT, 11 BPWA presented with general cognitive skills (e.g., attention, memory, executive functions, and visuospatial skills) that were within normal limits or mildly impaired. According to the BNT and BAT scores, BPWA presented with lexical deficits in both English and Spanish. Nine BPWA presented with diagnostic scores that were generally higher in English compared to Spanish, two BPWA presented with diagnostic scores that were generally higher in Spanish compared to English and one BPWA appeared to be more balanced in conversation but his testing battery was incomplete. See Table 3 for BPWA diagnostic scores.

### 2.3. Experiment Tasks

Participants completed one language control task, one semantic control task and one nonverbal task presented on a laptop using E-Prime 2.0 [70]. The order of the tasks was counterbalanced across participants and each experiment began with a practice session. First, the experiment was explained to the participant using hardcopy images. Then, the images were presented on the computer on a Power Point (Microsoft) presentation, which was followed by a practice set of stimuli presented on E-Prime with timing set to match the experiment. After the participant demonstrated appropriate understanding of the task, the experiment was administered. For all tasks, accuracies and response times (RTs) were recorded. Frequencies for all verbal stimuli items were calculated using the Cross-Linguistic Easy-Access Resource for Phonological and Orthographic Neighborhood Densities database (Clearpond) [71]. All items were matched on frequency for within- and between-language comparisons, cognates were excluded from experimental stimuli data sets, and each word only appeared within one word-pair in each experiment.

#### 2.3.1. Language Control Task

**Figure 1:**
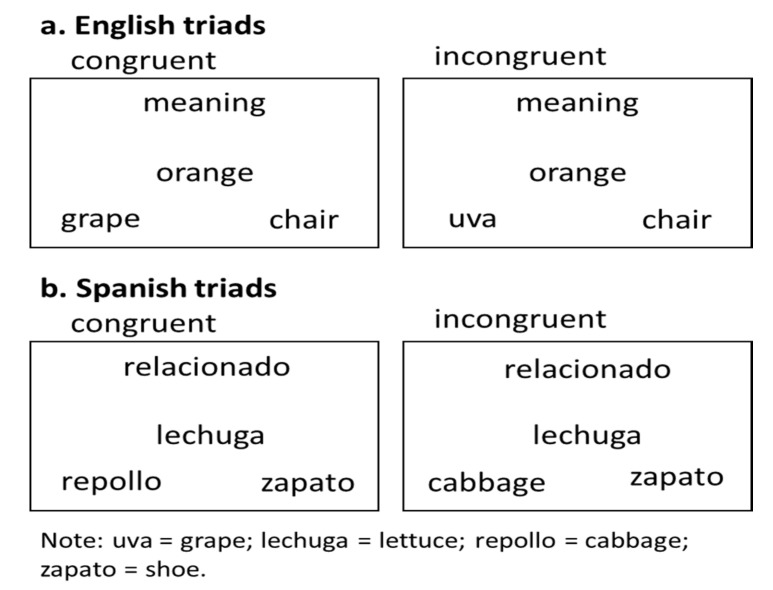
Language Control Task: (**a**) English congruent and incongruent triads; (**b**) Spanish congruent and incongruent triads.

The language control task was designed to determine the effect of how participants manage language control (i.e., within-language control vs. between-language control). All trials begin with a cue in English (“meaning”) or Spanish (“relacionado”) that appears on the top of the screen for 500 ms. The cue is followed by stimuli words presented as a triad: the given word is located in the middle of the screen and target and distractor words are located in the lower left and right corners of the screen. The location of the target and distractor words is pseudorandomized. The triad remains on screen for 3000 ms and is followed by a fixation (1000 ms). Participants are instructed to read the given word and identify the best semantic match from the two words located in the lower right and left corners of the screen. Congruent trials include within-language word-pairs and incongruent trials include between-language word-pairs. The incongruent condition is expected to elicit longer RTs and/or lower accuracy compared to the congruent condition because it requires more effort to resolve the between-language conflict to make the correct response. This task has been used previously and indexes resistance to distractor interference [58]. A total of 160 trials are presented across 5 blocks that each include 32 trials (16 congruent and 16 incongruent). Participants respond with a button press. The word in the lower left corner is mapped to a “1” on the keyboard and the word in the lower right corner is mapped to a “2” on the keyboard. See Figure 1.

#### 2.3.2. Semantic Control Task

**Figure 2:**
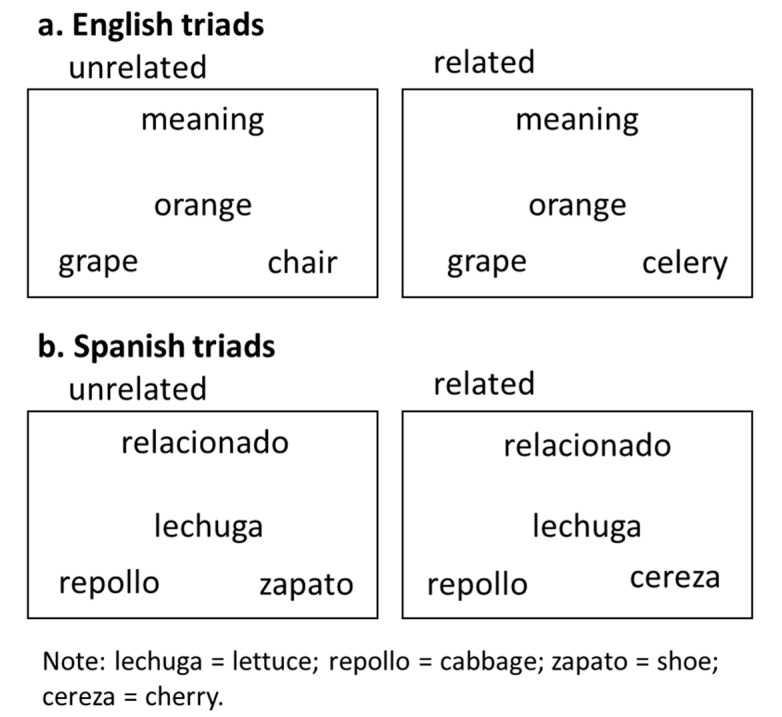
Semantic Control Task: (**a**) English unrelated and related triads; (**b**) Spanish unrelated and related triads.

The semantic control task was designed to determine the effect of how participants manage semantic interference. Similar to the language control task, each trial begins with an English or Spanish cue (500 ms). The words appear as a triad (given word, target word and distractor word) that are on screen for 3000 ms, and the location of the target and distractor words is pseudorandomized. The trial ends with a fixation (1000 ms). Like the language control task, participants are instructed to read the given word and identify the best semantic match from the two words located in the right and left corners of the screen. Distinct from the language control task, all trials are within-language (Spanish or English) and the task includes unrelated and related trials. On unrelated trials, the distractor item is unrelated to the given and target words and on related trials the distractor item is semantically related to the given word but not as closely as the target word. Because the semantically related distractor evokes semantic interference and requires more effort to suppress, the related condition is expected to elicit longer RTs and/or longer accuracy compared to the unrelated condition. A total of 160 trials are presented across 5 blocks that each include 32 trials (16 related and 16 unrelated), and the word in the lower left corner is mapped to a “1” on the keyboard and the word in the lower right corner is mapped to a “2” on the keyboard. See Figure 2.

#### 2.3.3. Nonverbal Control Task

**Figure 3:**
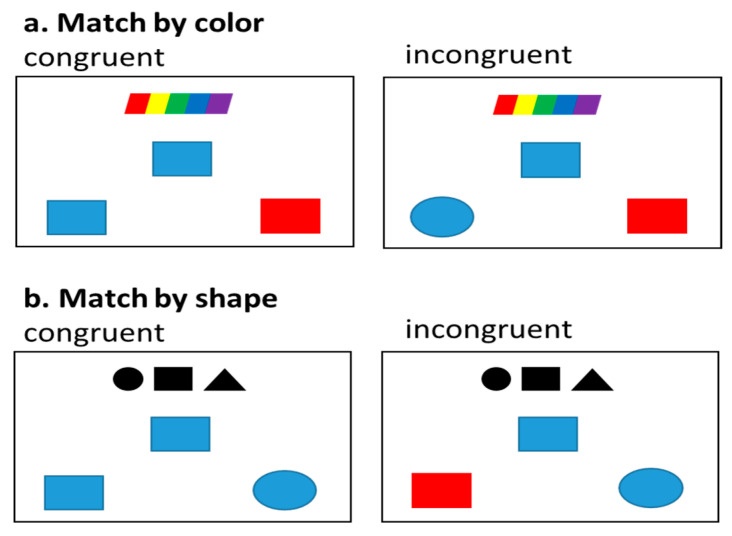
Nonverbal Control Task: (**a**) Match by color congruent and incongruent triads; (**b**) Match by shape congruent and incongruent triads.

The nonverbal control task was designed to determine the effect of how participants access the target dimension (i.e., color or shape) while simultaneously inhibiting the non-target dimension (i.e., color or shape). The experimental design is similar to the verbal tasks. First, a cue appears on the top of the screen (1000 ms) that consists of either three small black shapes that indicate to matching by shape or a small rainbow pattern that indicates matching by color. Following the cue, the given item, located in the middle of the screen, and the target and distractor items, located in the lower left and right corners of the screen, appear for 3000 ms. The location of target and distractor items is pseudorandomized, and the triad is followed by a fixation (500 ms). Stimuli include shapes (circles, squares, and triangles) and colors (red, blue, green). Participants were instructed determine which of the bottom shapes (i.e., the target or distractor) match the given item based on the cue type. This task has been shown to elicit the congruency effect and examine resistance to distractor interference [58,62]. A total of 160 trials are presented across 5 blocks that each include 32 trials. Each block consists of 16 color trials (8 congruent and 8 incongruent) and 16 shape trials (8 congruent and 8 incongruent). The targets and distractors are mapped to “1” and “2” keyboard button press responses. Congruent trials include stimuli that are all the same shape or all the same color; therefore, the non-target dimension does not require inhibition. Incongruent trials include color and shape stimuli that need to be inhibited. For example, on an incongruent shape trial that includes a blue square as the given item, the target is a red square and distractor is a blue circle. In order to identify the target, the red color of the target must be inhibited in order to select by shape. See Figure 3.

## 3. Results

### 3.1. Do AMBA and BPWA Exhibit the Congruency Effect on Language Control and Nonverbal Control Tasks and the Relatedness Effect on the Semantic Control Task?

#### 3.1.1. Data Analysis

AMBA and BPWA data was analyzed separately. For all response time (RT) statistical models, the data set only included accurate responses and responses below 200 ms or above 2.5 SDs from the mean were excluded. For all analyses, an alpha level of *p* < 0.05 was used. Language control task: For each group, two 2 × 2 repeated measures ANOVAs examining the effect of language Spanish/English) and presence of congruency (congruent/incongruent) were performed for percent accuracy and RT. Semantic control task: For each group, two 2 × 2 repeated measures ANOVAs examining the effect of language (Spanish/English) and presence of relatedness (unrelated/related) were performed for percent accuracy and RT. Nonverbal control task: For each group, two 2 × 2 repeated measures ANOVAs examining the effect of dimension (color/shape) and presence of congruency (congruent/incongruent) were performed for percent accuracy and RT. See Table 4 for AMBA and BPWA response times and percent accuracy for each task.

#### 3.1.2. Language Control Task

For AMBA, within-subjects analyses revealed no significant main effect of congruency on accuracy (*F*(1, 19) = 0.073, *p* = 0.79, η_p_^2^ = 0.004), but RT was significant (*F*(1, 19) = 5.04, *p* < 0.05, η_p_^2^ = 0.21), indicating that AMBA were faster on congruent trials compared to incongruent trials. There was no significant main effect of language on accuracy (*F*(1, 19) = 1.50, *p* = 0.23, η_p_^2^ = 0.07) or RT (*F*(1, 19) = 1.19, *p* = 0.28, η_p_^2^ = 0.06), indicating no difference in accuracy and RT for Spanish and English. The language by congruency interaction was not significant on accuracy (*F*(1, 19) = 0.15, *p* = 0.70, η_p_^2^ = 0.008) but did show significance on RT (*F*(1, 19) = 7.51, *p* < 0.05, η_p_^2^ = 0.28). Post hoc LSD pairwise comparisons revealed that the congruency effect was observed for English (*p* < 0.01) but not for Spanish (*p* = 0.35).

**Figure 4:**
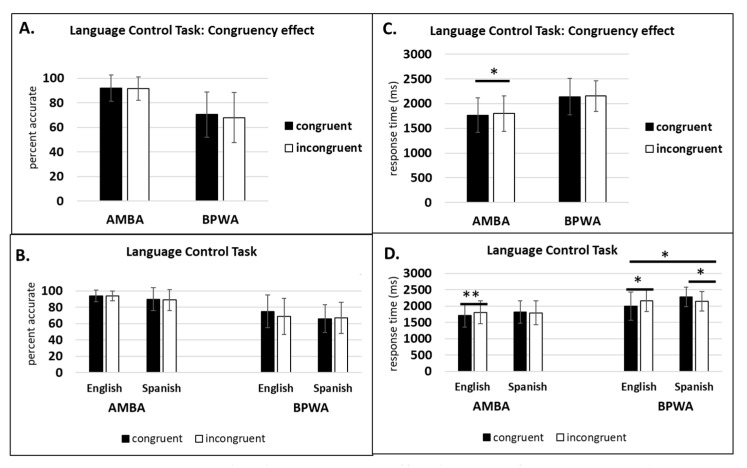
Language Control Task: (**A**) congruency effect percent accurate; (**B**) congruency effect percent accurate by group and language; (**C**) congruency effect response time (ms); (**D**) congruency effect response time (ms) by group and language. * *p* < 0.05, ** *p* < 0.01.

For BPWA, within-subjects analyses revealed no significant main effect of congruency on accuracy (*F*(1, 11) = 0.84, *p* = 0.37, η_p_^2^ = 0.07) or RT (*F*(1, 11) = 0.09, *p* = 0.75, η_p_^2^ = 0.009), indicating that BPWA were not more accurate or faster on congruent trials compared to incongruent trials. There was no significant main effect of language on accuracy (*F*(1, 11) = 1.93, *p* = 0.19, η_p_^2^ = 0.15), but the language effect on RT was significant (*F*(1, 11) = 5.88, *p* < 0.05, η_p_^2^ = 0.34), indicating that BPWA were faster on English trials compared to Spanish trials. The language by congruency interaction was not significant for accuracy (*F*(1, 11) = 2.59, *p* = 0.13, η_p_^2^ = 0.19) but did show significance for RT (*F*(1, 11) = 17.22, *p* < 0.01, η_p_^2^ = 0.61). Post hoc LSD pairwise comparisons revealed that for English, the congruency effect was observed (*p* < 0.05) and for Spanish, the reverse congruency effect was observed (*p* < 0.05). See Figure 4.

#### 3.1.3. Semantic Control Task

For AMBA, within-subjects analyses revealed a significant main effect of relatedness on accuracy (*F*(1, 19) = 29.05, *p* < 0.001, η_p_^2^ = 0.60) and RT (*F*(1, 19) = 21.39, *p* < 0.001, η_p_^2^ = 0.53), indicating that AMBA were more accurate and faster on unrelated trials compared to related trials. There was no significant main effect of language on accuracy (*F*(1, 19) = 0.98, *p* = 0.33, η_p_^2^ = 0.04) or RT (*F*(1, 19) = 2.58, *p* = 0.12, η_p_^2^ = 0.12), indicating no difference in accuracy and RT for Spanish and English. The language by congruency interaction was not significant on accuracy (*F*(1, 19) = 0.02, *p* = 0.87, η_p_^2^ = 0.001) or RT (*F*(1, 19) = 0.02, *p* = 0.87, η_p_^2^ = 0.001).

**Figure 5:**
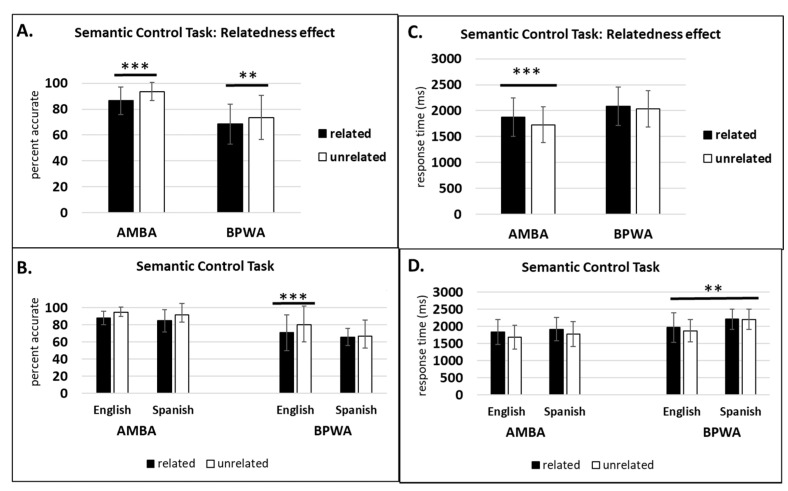
Semantic Control Task: (**A**) relatedness effect percent accurate; (**B**) relatedness effect percent accurate by group and language; (**C**) relatedness effect response time (ms); (**D**) relatedness effect response time (ms) by group and language. ** *p* < 0.01, *** *p* < 0.001.

For BPWA, within-subjects analyses revealed a significant main effect of relatedness on accuracy (*F*(1, 11) = 13.83, *p* < 0.01, η_p_^2^ = 0.55) but not on RT (*F*(1, 11) = 1.91, *p* = 0.19, η_p_^2^ = 0.14), indicating that BPWA were more accurate on unrelated trials compared to related trials. There was no significant main effect of language on accuracy (*F*(1, 11) = 3.03, *p* = 0.10, η_p_^2^ = 0.23), whereas RT was significant (*F*(1, 11) = 14.79, *p* < 0.01, η_p_^2^ = 0.57), indicating that BPWA were faster on English trials compared to Spanish trials. The language by congruency interaction was significant for accuracy (*F*(1, 11) = 5.25, *p* < 0.05, η_p_^2^ = 0.32) but not RT (*F*(1, 11) = 3.97, *p* = 0.07, η_p_^2^ = 0.26). Post hoc LSD pairwise comparisons revealed that the relatedness effect was observed for English (*p* < 0.001) but not for Spanish (*p* = 0.57). (See Figure 5.)

#### 3.1.4. Nonverbal Control Task

For AMBA, within-subjects analyses revealed a significant main effect of congruency on accuracy (*F*(1, 19) = 9.18, *p* < 0.01, η_p_^2^ = 0.32) and RT (*F*(1, 19) = 32.09, *p* < 0.001, η_p_^2^ = 0.62), indicating that AMBA were faster and more accurate on congruent trials compared to incongruent trials. There was no significant main effect of dimension on accuracy (*F*(1, 19) = 0.08, *p* = 0.77, η_p_^2^ = 0.004), but the dimension effect on RT was significant (*F*(1, 19) = 26.30, *p* < 0.001, η_p_^2^ = 0.58), indicating that AMBA were faster on color trials compared to shape trials. The dimension by congruency interaction was not significant on accuracy (*F*(1, 19) = 0.09, *p* = 0.76, η_p_^2^ = 0.005) or RT (*F*(1, 19) = 0.43, *p* = 0.51, η_p_^2^ = 0.02).

**Figure 6:**
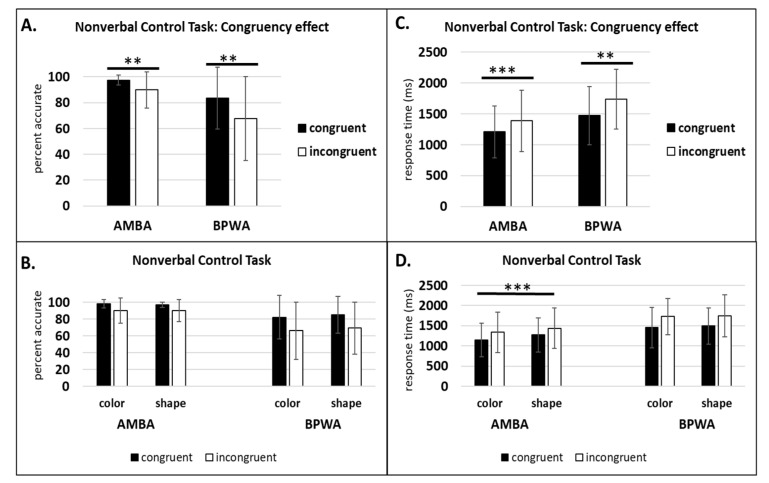
Nonverbal Control Task: (**A**) congruency effect percent accurate; (**B**) congruency effect percent accurate by group and language; (**C**) congruency effect response time (ms); (**D**) congruency effect response time (ms) by group and language. ** *p* < 0.01, *** *p* < 0.001.

For BPWA, within-subjects analyses revealed a significant main effect of congruency on accuracy (*F*(1, 11) = 9.89, *p* < 0.01, η_p_^2^ = 0.47) and RT (*F*(1, 11) = 16.13, *p* < 0.01, η_p_^2^ = 0.61), indicating that similar to AMBA, BPWA were faster and more accuracy on congruent trials compared to incongruent trials. In contrast to AMBA, BPWA showed no significant main effect of dimension on accuracy (*F*(1, 11) = 0.07, *p* = 0.78, η_p_^2^ = 0.007) or RT (*F*(1, 11) = 0.11, *p* = 0.75, η_p_^2^ = 0.01), indicating no difference in performance for color and shape. In line with AMBA, the dimension by congruency interaction was not significant for accuracy (*F*(1, 11) = 0.002, *p* = 0.96, η_p_^2^ = 0) or RT (*F*(1, 11) = 06, *p* = 0.81, η_p_^2^ = 0.006). See Figure 6.

### 3.2. Is the efficiency of Inhibition on the Language Control, Semantic Control and Nonverbal Control Tasks Different between Groups?

#### 3.2.1. Data Analysis

In order to examine the amount of conflict required to complete each task, conflict magnitudes were calculated for accuracy and RT for each task. This metric is used to capture the amount of control required to inhibit task irrelevant information in order to respond to the target. For the nonverbal control task and verbal control task, the conflict magnitude for accuracy is calculated by subtracting incongruent trials from congruent trials (i.e., congruent—incongruent) and for RT, congruent trials are subtracted from incongruent (i.e., incongruent—congruent). For the semantic control task, the conflict magnitude for accuracy is calculated by subtracting related trials from unrelated trials (i.e., unrelated—related) and for RT, unrelated trials are subtracted from related trials (i.e., related—unrelated).

To examine how AMBA and BPWA manage the control required to complete each task, two 2 × 3 repeated measures ANOVAs were performed to examine the effect of group (AMBA and BPWA) as the between-subjects factor and task (language control, nonverbal control, and semantic control) as the within-subject factor on conflict magnitude for accuracy and RT.

#### 3.2.2. Conflict Magnitudes

**Figure 7:**
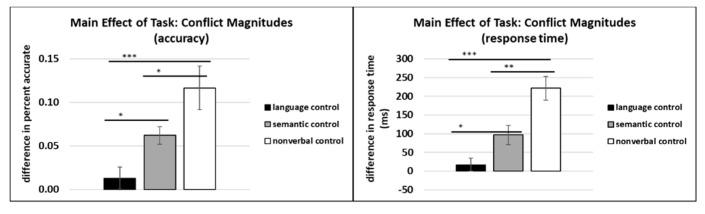
Conflict magnitude across tasks for accuracy and response time. * *p* < 0.05, ** *p* < 0.01, *** *p* < 0.001.

Results revealed no significant main effect of group for accuracy (*F*(1, 30) = 1.53, *p* = 1.53, η_p_^2^ = 0.04) or RT (*F*(1, 30) = 0.19, *p* = 0.66, η_p_^2^ = 0.006). However, there was a significant main effect of task on accuracy (*F*(2, 30) = 8.86, *p* < 0.001, η_p_^2^ = 0.38) and RT (*F*(2, 30) = 18.54, *p* < 0.001, η_p_^2^ = 0.57). Post hoc LSD pairwise comparisons revealed that the language control task conflict magnitude was significantly smaller than the semantic control task conflict magnitude for accuracy (*p* < 0.05) and RT (*p* < 0.05); the language control task conflict magnitude was also significantly smaller than the nonverbal control task conflict magnitude for accuracy (*p* < 0.001) and RT (*p* < 0.001); and the semantic control task conflict magnitude was significantly smaller than the nonverbal control task conflict magnitude for accuracy (*p* < 0.05) and RT (*p* < 0.01). See Figure 7.

**Figure 8:**
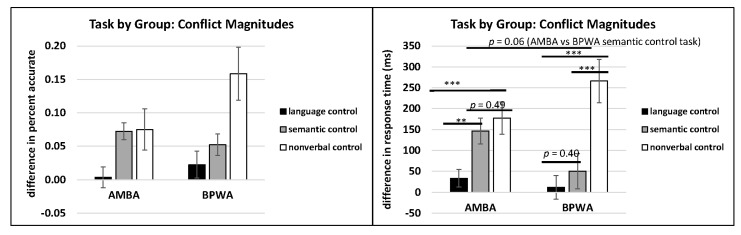
Conflict magnitude by group and task for accuracy and response time. ** *p* < 0.01, *** *p* < 0.001.

The task by group interaction was not significant for accuracy (*F*(2, 29) = 2.17, *p* = 0.13, η_p_^2^ = 0.13) but was significant for RT (*F*(2, 28) = 3.14, *p* = 0.05, η_p_^2^ = 0.18). Post hoc LSD pairwise comparisons revealed that AMBA conflict magnitude on the language control task was significantly smaller than on the nonverbal control task (*p* < 0.001) and on the semantic control task (*p* < 0.01), but there was no difference in conflict magnitude between the nonverbal task and the semantic task (*p* = 0.49). In contrast, BPWA conflict magnitude on the language control task was not significantly different than on the semantic control task (*p* = 0.40), but was significantly smaller than on the nonverbal control task (*p* < 0.001); meanwhile, the conflict magnitude on the nonverbal control task was significantly bigger than on the semantic control task (*p* < 0.001). See Figure 8.

To follow up and identify possible group differences on each task, LSD pairwise comparisons were examined. Results revealed no group differences on the language control task (*p* = 0.36) or on the nonverbal control task (*p* = 0.18); however, on the semantic control task, a group difference was approaching significance (*p* = 0.06) with BPWA presenting with a smaller conflict magnitude compared to AMBA.

### 3.3. Follow-up Analyses

**Figure 9:**
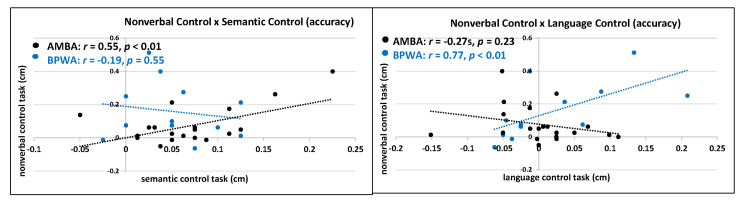
Correlations between control task conflict magnitudes for AMBA and BPWA.

For each group (AMBA and BPWA) correlations (Pearson and Spearman, respectively) were conducted to examine the relationship of control across tasks: (1) language control task and nonverbal control task; (2) language control and semantic control; and (3) semantic control task and nonverbal control task. Results revealed that, for AMBA, there was no significant correlation between language control and nonverbal control for accuracy (*r*(20) = −0.27, *p* = 0.23) or RT (*r*(20) = 0.16, *p* = 0.49) and no significant correlation between language control and semantic control for accuracy (*r*(20) = −0.23, *p* = 0.31) or RT (*r*(20) = −0.18, *p* = 0.42), whereas semantic control and nonverbal control were significantly related for accuracy (*r*(20) = 0.55, *p* < 0.01) but not for RT (*r*(20) = 0.13, *p* = 0.58). These AMBA results indicate that as it becomes more difficult to manage semantic control, it is more difficult to manage nonverbal control. For BPWA, there was a significant correlation between language control and nonverbal control for accuracy (*r*(12) = 0.77, *p* < 0.01) but not for RT (*r*_s_(12) = 0.25, *p* = 0.45); no significant correlation between language control and semantic control for accuracy (*r*_s_ (12) = −0.41, *p* = 0.18) or RT (*r*_s_ (12) = −0.31, *p* = 0.32); and no significant correlation between semantic control and nonverbal control for accuracy (*r*_s_ (12) = −0.19, *p* = 0.55) but not RT (*r*_s_ (12) = 0.08, *p* = 0.81). BPWA results indicate that as it becomes more difficult to manage language control, it is more difficult to manage nonverbal control. See Figure 9.

## 4. Discussion

The purpose of this study was to examine the relationship between language control, semantic control, and nonverbal control. AMBA and BPWA completed three control tasks, and each task examined resistance for distractor interference [44] and was designed to test language control, semantic control, or nonverbal control. The language control task and nonverbal control task elicited interference and conflict resolution, referred to as the congruency effect (lower accuracy and/or slower response times on the incongruent condition compared to the congruent condition), and the semantic control task elicited semantic interference, referred to as the relatedness effect (lower accuracy and/or slower response times on the related condition compared to the unrelated condition).

The first research question investigated whether AMBA and BPWA would show the congruency effect and relatedness effect. Based on previous work showing that people without aphasia exhibit slower RTs and/or lower accuracy on incongruent trials compared to congruent trials on verbal and nonverbal control tasks and semantic interference [17,28,48,49], it was hypothesized that AMBA would demonstrate the congruency effect on the language control task and nonverbal control task and the relatedness effect on the semantic control task. AMBA results supported the expected outcomes, indicating that it was more difficult to inhibit and resolve conflict on the incongruent and related conditions compared to the congruent and unrelated conditions. The language control task and nonverbal task have been used before [58,62,72,73], so predictions were clear. The semantic control task was novel yet elicited a strong effect of semantic interference, offering validation for the task.

Based on previous work showing that BPWA are susceptible to nonverbal control and semantic interference [29,57,58,59,60,61,65], it was hypothesized that BPWA would show the congruency effect on the nonverbal task and the relatedness effect on the semantic task. In line with predictions, BPWA did present with lower accuracies and/or longer RTs on the incongruent and related conditions compared to the congruent and unrelated conditions. Due to conflicting findings on bilingual patient performance on language control tasks [57,60,74,75], it was unclear whether BPWA would exhibit the congruency effect on the language control task. BPWA have been shown to present with cross-language intrusions and language switching impairments [29,76,77,78]. Further, previous work using the same language control task in the present study revealed that BPWA did not show the congruency effect on that task [58]. However, the different outcomes may highlight the importance of aphasia severity and how it influences language processing. Patients with less severe aphasia are better at processing language than patients with more severe aphasia [79]. On average, BPWA in the current study present with less severe aphasia deficits than BPWA in Gray and Kiran [58]. Although the language control task is designed to examine how bilinguals manage two languages, accessing semantic representations and retrieving word forms is an integral part of the task. Therefore, for patients who present with more severe aphasia deficits, the task may be capturing lexical access, rather than language control.

For the second research question, conflict magnitude was used to index efficiency of inhibition for each group and task. BPWA were expected to be less efficient at inhibiting and resolving conflict on all tasks compared to AMBA. The only group difference observed was that BPWA exhibited a smaller conflict magnitude on the semantic control task compared to AMBA. At first glance, this finding is counterintuitive. Typically, patient groups that present with impaired interference control exhibit larger conflict magnitudes and conflict ratios (indicative of less efficient control) compared to their age-matched counterparts [17,29,57,60,62]. However, this does not mean that the smaller conflict ratio for BPWA than AMBA found in this study represents more efficient control for the BPWA than the AMBA. BPWA exhibited a significant relatedness effect, informing us that they are susceptible to semantic interference. Thus, a small conflict ratio may be indicative of overall slowed processing or less efficient inhibitory control. In 2016, Gray and Kiran [59] examined language control by asking AMBA and BPWA to match within- and between-language word-pairs that were semantically related or semantically unrelated. Both groups demonstrated positive effects of semantic facilitation. However, compared to AMBA, BPWA presented with diminished effects of semantic control, similar to the current findings.

Within-group conflict magnitude patterns were also examined. AMBA exhibited more conflict (i.e., greater conflict magnitudes) on the semantic control task compared to the language control task, indicating that it is more difficult to inhibit semantically related distractors compared to managing two languages. Logically, we can hypothesize that because language control is intrinsic to a bilingual’s everyday experience, inhibiting a non-target language may require less effort than inhibiting a semantic distractor. Proficient bilinguals frequently and seamlessly switch between languages, it is a natural process [80,81]. It has also been shown that items with a higher frequency are more frequently switched [82] and that codeswitching is a natural part of a bilingual’s experience [83]. Since high frequent words were used in this study, those word types may have facilitated language management (resulting in small conflict magnitudes) on the language control task. Notably, on the semantic control task, in addition to high frequent word types, because the “related” distractor word is semantically related to the given word and target word, its activation is increased, which requires more effort to suppress, whereas on the language control task, the distractor words are not semantically related to the given word and target word. Thus, the language control task appears to be less demanding than the effortful semantic control task.

BPWA data revealed no difference between language control and semantic control conflict magnitudes. When these data are considered in relation to the previous finding (that BPWA exhibit weaker effects of semantic interference compared to AMBA), it could be that the primary deficit is not language control, per se, but semantic control. Aphasia results in impaired access to lexical representations. While BPWA may present with deficits in language control as evidenced by pathological code-switching or cross-language intrusion errors, these deficits are typically exhibited by patients with subcortical lesions [84,85,86]. BPWA in the current study present with left hemisphere cortical lesions so pathological language switching is not expected. BPWA also exhibit the congruency effect on the language control task, suggesting that the lack of difference between language control and semantic control conflict magnitude lies within semantic control deficits, rather than language control impairment.

Finally, the possible overlap between these three types of control was examined. AMBA findings revealed a significant correlation between semantic control and nonverbal control, indicating that better semantic control is related to better nonverbal control. These findings align with Calabria et al. [29]. Calabria and colleagues [29] used conflict magnitudes to examine the relationship between the semantic interference effect from a blocked naming task and flanker task conflict in AMBA and BPWA. Results identified a positive correlation between these variables, suggesting a possible overlap between semantic and nonverbal control mechanisms for the non-dominant language only. In contrast to the current findings, the Gray and Kiran [58] data set previously discussed found that AMBA demonstrated a significant correlation between language control and nonverbal control. Reflecting upon this, perhaps significant and non-significant correlations across domains are a function of bilingual life experience [87,88]. Prior and Gollan [88] asked three participant groups—(1) Spanish-English bilingual adults who frequently switched languages in their daily lives, (2) Chinese-English bilingual adults who did not frequently switch languages in their daily lives, and (3) monolingual adults—to complete a task-switching and language-switching task. Results revealed that on the language-switching task, the Spanish-English bilinguals outperformed the Chinese-English bilinguals, and that the Chinese-English bilinguals did not exhibit a task-switching advantage compared to the monolingual group. These findings underscore the importance of a more thorough investigation regarding bilingual language exposure and language use across the lifetime when interpreting results that capture domain general and domain specific relationships.

BPWA correlation results revealed a significant relationship between language control and nonverbal control, indicating that for BPWA, decreased efficiency at managing language control is associated with decreased efficiency at managing nonverbal control. At first glance, this result is surprising because it suggests a possible overlap between language control and nonverbal control mechanisms not observed in AMBA; however, upon closer inspection, it appears that it might capture the multi-step process intrinsic to the language control task. Managing semantic processes and language comprehension activates language centers, as well as areas responsible for domain general cognitive processing [89,90,91]. There is evidence that in addition to language processing, the left inferior frontal gyrus is recruited to complete semantically ambiguous tasks [92] and verbal and nonverbal cognitive tasks [93], including task selection [94], suggesting that that it is connected to a domain general cognitive network. To complete the language control task, first, BPWA must engage their impaired semantic system to read words and access the semantic representations. It may contribute to an increase in cognitive load, forcing BPWA to rely more heavily on domain general processes to complete the semantic task, which is considered automatic processing for AMBA. For BPWA, the conflict magnitude on the language control task may be capturing effects of domain general cognitive control as a function of increased cognitive load, rather than domain general cognitive control as a function of language control.

A few more observations in the data are worth noting. On the language control task, BPWA exhibited the reverse congruency effect for Spanish, meaning that they were faster on the Spanish incongruent condition compared to the Spanish congruent conditions. This data trend was unexpected. The incongruent condition requires the manipulation of two languages, which is more effort than reading words within one language. However, this patient group was primarily English dominant and educated in English. It could be that the English on the Spanish incongruent trials primed the word-match, resulting in fast response times. Another point of interest was that on the nonverbal task, AMBA were faster on the color trials compared to shape trials. The dimension of color is easier to manage than shape, thus it is hypothesized that to inhibit color when the target is shape would be more difficult than vice versa [72], and the data in the present study support this notion. Pertinent to the current study is that BPWA findings showed no difference between color and shape trials for accuracy or response time, suggesting that although BPWA exhibited the congruency effect on the nonverbal control task and there was no difference between AMBA and BPWA conflict magnitudes for the nonverbal control task, upon closer inspection, the nuances in the data reveal some level of abnormal management of nonverbal stimuli for BPWA.

Another observation worth mentioning is that AMBA and BPWA did not exhibit the congruency effect and relatedness effect across English and Spanish consistently. For example, on the language control task, AMBA and BPWA exhibited the congruency effect only for English trials. Of the 20 AMBA participants, 18 had acquired Spanish at birth and were more confident in Spanish. However, according to the Language Ability Rating on the Language Use Questionnaire, at the time of participation in this study, 10 of the 20 participants were highly proficient in Spanish and English or were English dominant and reported more exposure to English in their daily lives. The majority of BPWA who participated in this study acquired Spanish at birth, and approximately half were more confident in Spanish. Similar to AMBA, according to the Language Ability Rating on the Language Use Questionnaire, at the time of testing (i.e., after their injury), the majority of BPWA were balanced bilinguals or English dominant and reported more exposure to English in their daily lives. Even though the majority of AMBA and BPWA participants are stronger in English, it would be expected that participants would show the congruency effect in both Spanish and English. It could be that a combination of language dominance and current exposure is driving these findings and to better understand these data trends, future work should more closely examine bilingual lifestyle factors and how they impact language control.

On a related note, there are factors related to bilingualism that may affect post-stroke verbal and nonverbal performance. For instance, language proficiency, recovery patterns, and age of acquisition have been shown to interact with post-stroke language presentation. Addressing all of these factors are beyond the scope of the current study; however, they warrant acknowledgement for future research directions. Furthermore, aphasia severity also interacts with verbal and nonverbal performance and future studies should focus on its role in patient outcomes.

Finally, when researchers examine control mechanisms in AMBA and BPWA, it is important that the experimental tasks are chosen carefully. For example, the language control task employed in the current study and the Stroop task have both been used to investigate language control. Both tasks examine inhibitory control; however, according to Friedman and Miyake [44], they tap different types of inhibitory control. Specifically, the Stroop task examines prepotent response inhibition because it requires the inhibition of an automatic response (i.e., participants must say the color of ink while ignoring the written word), and the language control task examines resistance to distractor interference because non-target distractors are presented alongside the target items. Although these types of inhibition are related [44], they are distinct. Moreover, the Stroop task is a within-language task, whereas the language control task in the current study is a between-language task, offering the opportunity to examine bilingual language control.

## 5. Conclusions

The goal of this study was to better understand the relationship between language control, semantic control, and nonverbal control in bilingual aphasia. A consistent and persistent characteristic of aphasia is impaired lexical access. This disturbance may be the result of impaired activation of the target word, impaired inhibition of lexical competitors or a combination of these two factors. Lexical access also requires semantic control. When accessing a word, semantically related competitors must be inhibited so that the target is selected. BPWA must also manage language control. To speak, they must activate the target language while simultaneously suppressing the non-target language. There is also evidence that nonverbal control overlaps with language control to some extent. The current findings indicate that AMBA and BPWA exhibited significant effects of control across all tasks. The results also suggest that for AMBA, a greater effort is required to manage semantic interference and nonverbal control compared to language control, whereas for BPWA, this difference in effort to manage language control and semantic control was not observed. Additionally, AMBA results indicated an association between semantic control and nonverbal control, suggesting that these two types of control may overlap. BPWA data revealed a significant correlation between language control and nonverbal control mechanisms that may be reflective of increased cognitive load, rather than domain general cognitive control. The findings from this study contribute to our knowledge concerning BPWA verbal and nonverbal strengths and weaknesses, offering more information to inform how we approach language and cognitive rehabilitation for BPWA.

## Figures and Tables

**Table 1:** Language Use Questionnaire: Age-Matched Bilingual Adults.

AMBA	AoA		Lifetime Exposure		Confidence		Current Exposure		Family Proficiency		Education History		Language Ability Rating	
	E	S	E	S	E	S	E	S	E	S	E	S	E	S
**1**	5	0	59	41	75	90	ND	ND	50	92	83	17	97	83
**2**	6	0	51	49	47	67	71	29	25	67	72	28	80	60
**3**	14	0	41	59	51	100	66	34	92	100	50	50	100	100
**4**	0	15	100	10	100	10	100	10	100	0	100	0	100	20
**5**	25	0	20	76	21	95	50	50	33	100	0	100	80	91
**6**	2	0	75	25	100	57	89	11	92	67	72	28	100	69
**7**	12	0	38	63	19	100	74	26	25	100	33	67	94	100
**8**	14	0	21	79	47	100	97	3	33	100	33	67	80	91
**9**	13	0	93	8	47	100	84	16	25	100	11	78	97	100
**10**	38	0	8	92	12	100	25	75	8	100	11	111	60	100
**11**	11	0	55	45	58	100	93	7	58	100	61	39	100	94
**12**	17	0	30	70	5	100	49	51	17	100	50	50	66	86
**13**	0	5	89	11	96	66	97	3	17	100	100	0	100	54
**14**	5	0	68	33	90	98	74	26	50	100	100	0	100	100
**15**	23	0	10	90	8	100	27	73	0	100	0	100	66	100
**16**	16	0	34	66	49	100	26	74	25	100	33	67	77	100
**17**	18	0	27	73	30	100	32	68	25	100	0	100	63	100
**18**	13	0	12	89	21	100	6	94	25	100	8	92	46	100
**19**	18	0	4	96	11	100	60	40	0	100	8	92	51	100
**20**	12	0	21	76	28	93	66	34	0	100	0	100	86	100

Note: AMBA = age-matched bilingual adults, AoA = age of acquisition, E = English, S = Spanish, values are percentages.

**Table 2:** Language Use Questionnaire: Bilingual Persons with Aphasia.

BPWA	AoA		Lifetime Exposure		Confidence		Current Exposure		Family Proficiency		Education History		Pre-injury Language Ability Rating		Post-injury Language Ability Rating	
	E	S	E	S	E	S	E	S	E	S	E	S	E	S	E	S
**1**	10	0	57	38	60	48	96	4	100	100	50	50	100	70	80	60
**2**	9	0	63	33	88	85	28	72	42	93	83	17	91	67	60	46
**3**	5	0	67	28	88	69	64	36	33	100	83	17	100	100	67	37
**4**	13	0	40	55	64	100	98	2	87	100	67	33	97	97	63	46
**5**	50	0	38	63	2	97	0	100	0	100	100	0	40	100	20	60
**6**	25	0	10	83	9	100	42	58	25	100	17	83	100	100	32	60
**7**	0	0	91	6	100	0	100	0	ND	ND	100	0	100	20	40	20
**8**	7	0	54	41	83	96	50	50	25	100	83	17	100	100	63	60
**9**	0	3	77	48	100	42	100	0	100	33	78	22	100	71	47	20
**10**	7	0	62	33	83	100	87	13	83	100	44	56	100	100	91	80
**11**	4	0	49	46	79	94	96	4	42	75	58	42	100	100	37	66
**12**	0	12	97	0	97	23	100	0	100	13	79	21	97	40	77	20

Note: BPWA = bilingual person with aphasia, AoA = age of acquisition, E = English, S = Spanish, values are percentages.

**Table 3:** Language and Cognitive Diagnostic Testing.

Variable	BPWA1		BPWA2		BPWA3		BPWA4		BPWA5		BPWA6		BPWA7		BPWA8		BPWA9		BPWA10		BPWA11		BPWA12	
Age	57		48		27		51		52		65		42		65		52		61		30		54	
Sex	Female		Male		Male		Male		Male		Male		Male		Female		Female		Male		Male		Female	
Education	18		13		13		13		9		16		13		13		16		16		12		18	
Months post-injury	96		24		144		48		60		240		72		72		48		60		12		48	
Lesion region	LMCA		TBI		LMCA		LMCA		LMCA		LMCA		LMCA		LMCA		LMCA		LMCA		TBI		LMCA	
	En	Sp	En	Sp	En	Sp	En	Sp	En	Sp	En	Sp	En	Sp	En	Sp	En	Sp	En	Sp	En	Sp	En	Sp
Boston Naming Test	65%	17%	85%	38%	12%	3%	68%	30%	7%	57%	38%	58%	30%	3%	40%	18%	87%	32%	77%	42%	83%	DNT	98%	7%
Bilingual Aphasia Test Part B																								
receptive language composite	88%	76%	95%	93%	53%	51%	95%	90%	38%	69%	59%	61%	79%	21%	57%	73%	92%	86%	91%	66%	DNT	79%	95%	57%
expressive language composite	86%	54%	98%	85%	25%	0%	88%	66%	12%	88%	65%	83%	66%	0%	57%	18%	67%	72%	88%	77%	DNT	75%	100%	26%
Judgment of words/nonwords	93%	87%	100%	83%	83%	43%	97%	93%	53%	93%	90%	97%	97%	43%	93%	80%	93%	97%	70%	90%	DNT	80%	100%	73%
Bilingual Aphasia Test Part C																								
Translation	into E	into S	into E	into S	into E	into S	into E	into S	into E	into S	into E	into S	into E	into S	into E	into S	into E	into S	into E	into S	into E	into S	into E	into S
Word Recognition	100%	100%	100%	100%	60%	100%	100%	100%	60%	100%	100%	100%	80%	40%	80%	60%	100%	100%	100%	100%	DNT	DNT	100%	80%
Translation of Words	90%	40%	70%	80%	0%	50%	40%	70%	10%	40%	50%	20%	0%	10%	20%	60%	100%	60%	80%	80%	DNT	DNT	30%	50%
Translation of Sentences	61%	22%	72%	89%	0%	0%	67%	94%	0%	0%	61%	44%	0%	17%	0%	6%	67%	56%	61%	83%	DNT	DNT	0%	11%
Cognitive Linguistic Quick Test																								
Attention	Mild		WNL		WNL		WNL		Moderate		WNL		WNL		Mild		WNL		WNL		WNL		WNL	
Memory	Moderate		WNL		Moderate		Moderate		Severe		WNL		Moderate		Severe		Moderate		Mild		WNL		moderate	
Executive Functions	WNL		WNL		WNL		WNL		Severe		Moderate		Mild		Severe		WNL		Mild		WNL		WNL	
Language	Mild		WNL		Moderate		MILD		Severe		Mild		Moderate		Moderate		Mild		Mild		Mild		mild	
Visuospatial Skills	Mild		WNL		WNL		WNL		Severe		WNL		WNL		Mild		WNL		WNL		WNL		WNL	
Composite Severity	Mild		WNL		Mild		MILD		Severe		Mild		Mild		Moderate		Mild		Mild		WNL		Mild	
Clock Drawing	Mild		WNL		Severe		WNL		Severe		Mild		WNL		Moderate		Mild		Moderate		Mild		WNL	
Pyramids and Palm Trees																								
3 Pictures	87%		98%		88%		96%		54%		96%		75%		94%		90%		90%		DNT		100%	

Note: En = English; Sp = Spanish; into E= into English; into S = into Spanish; Bilingual Aphasia Test (BAT) receptive language composite score is an average of subtests: pointing, semicomplex commands, complex commands, semantic categories, synonyms, antonyms, semantic acceptiability; BAT expressive language composite scores is an average of subtests: series (automatics), object naming, and semantic opposites; WNL = within normal limits.

**Table 4:** Language Control Task, Semantic Control Task, and Nonverbal Control Task response time and percent accurate data for AMBA and BPWA.

Task	Condition	AMBA				BPWA			
		RT	SD	Acc	SD	RT	SD	Acc	SD
Language Control Task	English congruent	1717	358	94	7	1999	431	75	20
	Spanish congruent	1815	347	90	14	2282	297	66	17
	English incongruent	1807	351	94	6	2162	326	69	22
	Spanish incongruent	1791	365	89	13	2142	296	67	19
Semantic Control Task	English unrelated	1684	326	95	6	1867	362	80	20
	Spanish unrelated	1773	364	92	9	2204	345	68	15
	English related	1833	376	88	8	1963	371	71	21
	Spanish related	1915	357	85	13	2207	367	66	11
Nonverbal Control Task	color congruent	1147	416	98	5	1396	510	82	26
	shape congruent	1271	426	98	3	1428	483	85	22
	color incongruent	1335	503	90	15	1654	499	66	34
	shape incongruent	1437	497	90	13	1746	518	75	23

Note: AMBA = age matched bilingual adults; BPWA = bilingual persons with aphasia; RT = response time; acc = percent accurate; SD = standard deviation.
